# MGMT promoter methylation determined by HRM in comparison to MSP and pyrosequencing for predicting high-grade glioma response

**DOI:** 10.1186/s13148-016-0204-7

**Published:** 2016-05-05

**Authors:** Olivier J. Switzeny, Markus Christmann, Mirjam Renovanz, Alf Giese, Clemens Sommer, Bernd Kaina

**Affiliations:** Department of Toxicology, University Medical Center of the Johannes Gutenberg University Mainz, Obere Zahlbacher Strasse 67, 55131 Mainz, Germany; Department of Neurosurgery, University Medical Center of the Johannes Gutenberg University Mainz, Langenbeckstraße 1, 55131 Mainz, Germany; Department of Neuropathology, University Medical Center of the Johannes Gutenberg University Mainz, Langenbeckstraße 1, 55131 Mainz, Germany

**Keywords:** MGMT, Alkyltransferase, Brain tumors, Glioblastoma, Temozolomide, Promoter methylation, Epigenetic silencing, IDH1

## Abstract

**Background:**

The DNA repair protein O^6^-methylguanine-DNA methyltransferase (MGMT) causes resistance of cancer cells to alkylating agents and, therefore, is a well-established predictive marker for high-grade gliomas that are routinely treated with alkylating drugs. Since MGMT is highly epigenetically regulated, the MGMT promoter methylation status is taken as an indicator of MGMT silencing, predicting the outcome of glioma therapy. MGMT promoter methylation is usually determined by methylation specific PCR (MSP), which is a labor intensive and error-prone method often used semi-quantitatively. Searching for alternatives, we used closed-tube high resolution melt (HRM) analysis, which is a quantitative method, and compared it with MSP and pyrosequencing regarding its predictive value.

**Results:**

We analyzed glioblastoma cell lines with known MGMT activity and formalin-fixed samples from IDH1 wild-type high-grade glioma patients (WHO grade III/IV) treated with radiation and temozolomide by HRM, MSP, and pyrosequencing. The data were compared as to progression-free survival (PFS) and overall survival (OS) of patients exhibiting the methylated and unmethylated MGMT status. A promoter methylation cut-off level relevant for PFS and OS was determined. In a multivariate Cox regression model, methylation of MGMT promoter of high-grade gliomas analyzed by HRM, but not MSP, was found to be an independent predictive marker for OS. Univariate Kaplan–Meier analyses revealed for PFS and OS a significant and better discrimination between methylated and unmethylated tumors when quantitative HRM was used instead of MSP.

**Conclusions:**

Compared to MSP and pyrosequencing, the HRM method is simple, cost effective, highly accurate and fast. HRM is at least equivalent to pyrosequencing in quantifying the methylation level. It is superior in predicting PFS and OS of high-grade glioma patients compared to MSP and, therefore, can be recommended being used routinely for determination of the MGMT status of gliomas.

**Electronic supplementary material:**

The online version of this article (doi:10.1186/s13148-016-0204-7) contains supplementary material, which is available to authorized users.

## Background

Patients suffering from high-grade gliomas (notably glioblastoma multiforme, WHO grade IV) have a dismal prognosis (14.6 months median survival and a 2-year survival rate of 26 %) [1]. Their first-line therapy is based on the DNA alkylating agent temozolomide (Temodal*®*) and ionizing radiation [1, 2]. Temozolomide exerts its cytotoxic effect by the induction of O^6^-methylguanine, which represents an apoptosis-inducing DNA damage [3]. As second line drugs, DNA-chloroethylating agents (lomustine, nimustine, carmustine, and fotemustine) are being used, which cause toxicity via the formation of O^6^-chloroethylguanin and subsequently formed DNA interstrand crosslinks. In some studies, lomustine was used in combination with temozolomide in glioblastoma therapy [4–6]. The key node in defense against the cytotoxic DNA lesion O^6-^alkylguanine is O^6^-methylguanine-DNA methyltransferase (MGMT), a suicide repair enzyme that reverts the damage in a fast, stoichiometric, and error-free reaction [3, 7]. The expression of the repair protein and its suicide repair activity is inversely related to the killing response of glioblastoma cells in vitro [8] and the therapeutic outcome of glioblastoma therapy [9]. Thus, MGMT is an important predictive marker for high-grade gliomas [10, 11].

Since the determination of the MGMT activity relies usually on a radioactive assay, alternative techniques for detecting the MGMT status were established. These methods are based on the finding that the MGMT expression is highly regulated by MGMT promoter methylation [12, 13]. Most studies focused on methylation of two CpG islands positioned between −328 and −182 and between +28 and +117 relative to the ATG of the MGMT gene, which have been shown to provoke transcriptional silencing [14, 15]. Methylation of individual CpG sites in these islands of the MGMT promoter was shown to correlate with loss of MGMT protein expression in the tumor tissue [16]. MGMT promoter methylation is frequently analyzed via methylation specific PCR (MSP) [17] for which primer pairs flanking different CpG sites within the MGMT promoter are being used. The most commonly used primer was described by the Esteller group [18], which was used in a large number of studies. These studies revealed epigenetic silencing of MGMT in about 45 % of the cases and established a correlation between MGMT promoter methylation and patient’s overall survival (OS) and progression-free survival (PFS) [11, 16, 18–20].

MSP is a labor-intensive method that is often used in a non-quantitative way. It is error-prone as it requires the removal of the PCR product from the tube for further analysis, creating the potential for contamination. Searching for alternatives to MSP, we used a quantitative closed-tube real-time PCR with high resolution melt (HRM) analysis [21]. Using this method, we analyzed tumor specimens obtained from high-grade glioma patients and compared the data with MSP and also with pyrosequencing (PSQ). The data obtained were then related to progression-free survival (PFS) and overall survival (OS) of the patients. We found that HRM is clearly superior to MSP in discriminating between responders and non-responders. HRM was equal to PSQ, which is, however, more difficult to perform than HRM. We conclude that HRM is a fast, robust, and reliable method and excellent in predicting the outcome of glioma therapy.

## Methods

### Patients and treatments

Paraffin-embedded tumor samples were studied from 83 high-grade (WHO grade III and IV) glioma patients treated at the Neurosurgical Center at the University Medical Center of Mainz, Germany. Tumor specimens were obtained before radio-chemotherapy, formalin fixed, and paraffin embedded. DNA was extracted according to standard protocols. All patients provided written informed consent. The study was approved by the institutional ethics committee of the University Medical Center Mainz. Therapy regimen: All patients received combined radio-chemotherapy with temozolomide according to the EORTC regimen [1, 2]. In case of tumor progression, second-line therapy was administered, e.g., dose-dense temozolomide, CCNU, or bevacizumab. Two patients were lost to the follow-up after the first progress. The investigator performing the biochemical assays was blinded for all clinical information.

### Cell culture

Cells were cultured in DMEM (Gibco) supplemented with 10 % fetal calf serum (Gibco) and grown at 37 °C, 5 % CO_2_ atmosphere. DNA was isolated using phenol-chloroform followed by ethanol precipitation. The DNA samples were stored at −80 °C.

### DNA standards

Buccal DNA from a healthy donor was used to generate DNA standards. Fifty nanograms of DNA was used for whole genome amplification using the REPLI-g midi kit (Qiagen) to generate the unmethylated standard DNA. The reaction was performed according to the manufacturer’s instructions. An aliquot of 100 μg was in vitro methylated with 400 U SssI methylase and 640 μM SAM (NEB) according to the manufacturer’s instructions. After 4 h at 37 °C, additional SAM and 50 U of SssI methylase were added and incubated overnight at 37 °C to ensure complete methylation. Both methylated and unmethylated standard DNA were purified by phenol-chloroform extraction followed by ethanol precipitation, suspended in DNase-free water and stored at −80 °C.

### Bisulfite treatment

**Fig. 1:**
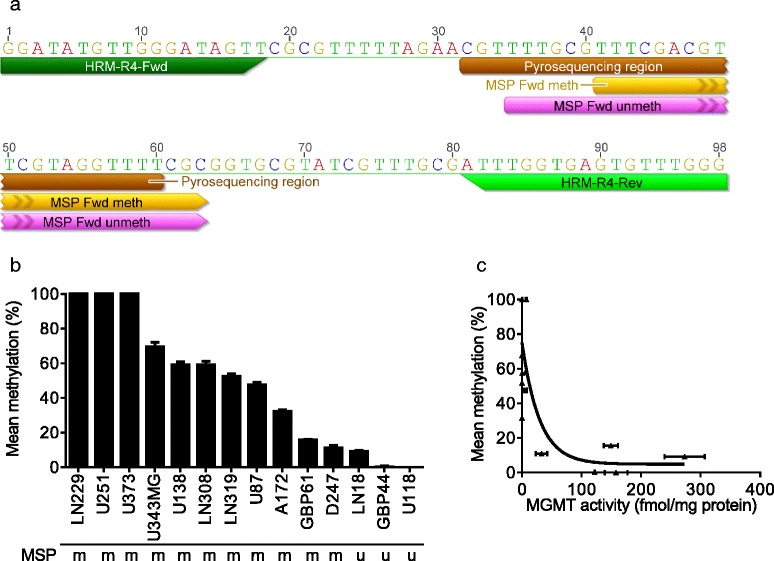
HRM analysis in glioma cell lines. **a** MGMT promoter methylation was analyzed by HRM, MSP, and PSQ. The regions in the MGMT promoter analyzed by each of the assays are shown. **b** MGMT promoter methylation of 14 GBM cell lines measured by HRM (in triplicates) and the corresponding MSP categorization (*m* methylated; *u* unmethylated). Ordinate shows the methylation level determined by HRM. **c** Relationship between MGMT suicide-enzyme activity and level of promoter methylation determined by HRM using the r4 primer in 14 GBM cell lines. A correlation between MGMT promoter methylation and MGMT activity was found (*r* = −0.69; *p* < 0.01)

Five hundred nanograms of DNA underwent bisulfite treatment using the EZ DNA methylation-kit (ZymoResearch) according to the manufacturer’s protocol to convert all unmethylated cytosine to uracil while leaving 5-methylcytosine unaltered, and was then eluted in 25 μl of DNase-free water. DNA methylation of the MGMT promoter was analyzed by MSP, pyrosequencing (PSQ), and HRM. The analyzed promoter sequences are shown in Fig. 1a.

### Analysis of the MGMT promoter methylation by MSP

For MSP of the MGMT promoter, we used primers previously described [18]. The method was carried out as described previously [22]. Classification was carried out binary, MGMT unmethylated and MGMT methylated, respectively.

### Analysis of the MGMT promoter methylation by high resolution melt (HRM) curve analysis

A search for CpG islands in the MGMT promoter was performed using the Geneious 6 software (Biomatters). For the HRM of the MGMT promoter, we used methylation independent primers (r4 fwd: 5′-GGATATGTTGGGATAGTT-3′ and r4 rev: 5′-CCCAAACACTCACCAAAT-3′) without a CpG site in it to avoid biased amplification (Additional file 1: Table S1). Primers were designed using the Pyromark assay Designer 2.0 (Qiagen). Region r2 and r4 include the binding sites of the MSP primers published by Esteller et al. [16]. PCR amplification and HRM analyses were performed using a CFX96 real-time PCR system (BioRad). Each PCR was performed in a final volume of 15 μl, containing 7.5 μl precision melt supermix (BioRad), 400 nM of each primer, and 20 ng of bisulfite-converted DNA (theoretical concentration presuming no loss of DNA during bisulfite modification). PCR amplification was performed with one step of 95 °C for 2 min, 45 cycles of 95 °C for 10 s, 54 °C for 30 s, and 72 °C for 15 s; followed by an HRM step of 95 °C for 30 s, 60 °C for 1 min, 70 °C for 10 s, and continuous acquisition to 90 °C at one acquisition per 0.2 °C. For cell lines, each reaction was performed in technical duplicates of biological triplicates, and in technical duplicates for the patient’s samples. Fully methylated and unmethylated bisulfite-converted DNA was mixed to obtain the following ratios of methylation: 2.1, 24.3, 46.4, 68.6, and 90.8 % (theoretically 0, 25, 50, 75, and 100 %) and were included in duplicates in each assay, as well as a non-template control and a genomic DNA control. Commercially available bisulfite-converted DNA standards (Qiagen) were analyzed together with our internal DNA standards. HRM data was analyzed using Bio-Rad Precision Melt Analysis software (BioRad), with output plots produced as normalized melting curves (Additional file 1: Figure S1A). Normalized relative fluorescence units (RFUs) were exported to Prism 6 (Graphpad). Area under the curve (AUC) was calculated, and the linear regression was used to interpolate the unknown samples from the standards. *R*^2^ was >0.98 (Additional file 1: Figure S1B).

### Analysis of the MGMT promoter methylation by pyrosequencing

The DNA methylation standards and patients DNA were analyzed by PSQ (Additional file 1: Figure S1C) to quantify their methylation content. PCR was performed using the PyroMark Q96 CpG MGMT kit (Qiagen) according to manufacturer’s instructions. The samples were then processed in the PyroMark Q96 ID instrument (Qiagen), and the obtained data were analyzed by PyroMark CpG Software. Patients were dichotomized upon a mean methylation level threshold of 8 % according to previous studies [23–25]. To further validate the methylation values of the HRM assay, we performed pyrosequencing for the whole HRM amplicon on a Pyromark Q24 advanced (Qiagen) for 38 patient samples and the DNA standards. The forward primer was also used as a sequencing primer (Additional file 1: Figure S2A and Table S1). The 38 methylation scores determined by HRM and pyrosequencing showed a high correlation (*r* = 0.926, *p* < 0.0001, Additional file 1: Figure S2B). The unmethylated DNA standard was methylated to an extent of 2.1 %, and the methylated DNA standard showed 90.8 % mean methylation at the MGMT promoter region. These values were taken for the linear regression analysis of data obtained by HRM (see above).

### MGMT activity assay

MGMT activity was measured for 14 GBM cell lines in triplicates using a protocol that has been published previously [26]. Briefly, the method is a radioactive assay in which tritium-labeled methyl group from the O^6^-position of guanine is transferred to the protein in the cell extract. Data were expressed as fmol of radioactivity transferred from ^3^H-labelled DNA to protein per milligram of protein within the sample.

### IDH1 mutation detection

The IDH1 mutational status was determined by immunohistochemistry using an anti-IDH1 R132H antibody (Dianova). We further validated the results by pyrosequencing (Additional file 1: Figure S1D) in all samples using primers published previously on the Pyromark Q96 ID instrument [27]. We excluded all IDH1-mutated patient samples from further analysis.

### Statistical analysis

Univariate survival and progression analyses and survival curves were estimated by the Kaplan–Meier method and compared using the log-rank test. Multivariate survival and progression analyses were performed using multiple Cox regression analysis. Two-tailed Spearman–Rho test was used to determine bivariate correlations between methylation status and patient characteristics. Two-tailed Pearson’s test was used to determine correlations of continuous methylation scores of the HRM and pyrosequencing assay. A *p* value <0.05 was considered statistically significant. An ROC curve was generated to graph the sensitivity and specificity of MSP, PSQ, and HRM status to predict OS ≥ 18 months and PFS ≥ 12 months. All statistics were computed using SPSS 23 (IBM) and plotted with Prism 6 (Graphpad).

## Results

The human MGMT gene was reported to harbor a CpG island of 762 bp in the promoter region (−531 to +231 from the ATG) containing 98 CpG sites [28]. We performed initially an in silico search for CpG islands 8 kb upstream and 1 kb downstream of the MGMT coding sequence that could be useful for HRM. Using Geneious software, we found a CpG island −729 to +461 from ATG, largely confirming the above study. Four primer sets (Additional file 1: Table S1) were analyzed as to their suitability for methylation analysis by HRM, using MGMT proficient (HaCaT) and MGMT deficient (LN229) cells. Primer pair r1 generated a 392 bp amplicon producing several melt peaks. It was therefore unsuitable for HRM analysis. Using primer pair r2 (covering the MSP reverse primer binding site), we observed only small differences in the methylation level between MGMT proficient versus deficient cells. The primer pairs r3 and r4 revealed extensive differences in the MGMT promoter methylation level and, therefore, were suitable for further analysis. The MGMT promoter methylation status was determined quantitatively by HRM in 14 GBM cell lines and compared with MSP (Fig. 1b). The regression analysis of promoter methylation determined by HRM and MGMT activity shows that the MGMT activity declines with increasing MGMT promoter methylation level, with r4 showing the best inverse correlation (Fig. 1c for r4, and Additional file 1: Figure S1E for r3). Therefore, primer pair r4 covering 12 CpGs, including the region that was analyzed using the MSP and PSQ assay (Fig. 1a), was used for our further studies with tumor tissue.

MGMT promoter methylation was analyzed in paraffinized tumor samples from 83 glioma patients. We found that MGMT promoter methylation was not associated with the patient’s age and sex (Table 1). MGMT promoter methylation was detected by MSP in 37.3 % of the cases, whereas HRM showed promoter methylation in 51.8 % and PSQ in 54.2 % of the samples (Table 1). Thus, HRM was similar to PSQ in detecting promoter methylation.

**Table 1:** Characteristics of patients and their MGMT promoter methylation status determined by HRM, MSP, and PSQ in 83 malignant gliomas, including 18 IDH1-mutated cases

Characteristics	*n*	HRM me (%)	MSP me (%)	PSQ me (%)	IDH1 mut (%)
All patients	83	51.8	37.3	54.2	21.7
Woman	28	50.0	39.3	53.6	21.4
Man	55	52.7	36.4	54.5	21.8
Age ≥70	27	44.4	25.9	44.4	0.0
Age <70	56	55.4	42.9	58.9	32.1
Grade III	23	73.9	56.5	69.6	69.6
Grade IV	60	43.3	30.0	48.3	3.3

Since IDH1-mutated tumors show a favorable patient survival and since the IDH1 status is considered to be an independent prognostic marker for WHO grade III gliomas [29], we decided to exclude IDH1-mutated samples from further analysis. IDH1 mutation was analyzed by immunohistochemistry using an anti-IDH1 R132H antibody and further confirmed by sequencing. The analysis revealed 18 of 83 analyzed tumors as IDH1 mutated. The IDH1 mutations were predominantly observed in grade III (88.9 %), but also in grade IV tumors (11.1 %) and a high correlation with MGMT promoter methylation was observed. Thus, 88.9 % of *IDH1*-mutated tumors displayed MGMT promoter methylation (Additional file 1: Table S2), confirming data in the literature [30, 31].

To determine an optimal cut-off value for discriminating between methylated and unmethylated MGMT promoter, ROC curves were plotted for 15 methylation cut-off scores (1–15 %) to identify the optimum cut-off level for the prediction of PFS ≥12 months and OS ≥18 months. The cut-off value of 5 % showed the largest AUC for both PFS (0.705) and OS (0.637) (Additional file 1: Table S3) confirming the suitability of a 5 % cut-off value for discriminating between the methylated and the unmethylated MGMT promoter.

**Fig. 2:**
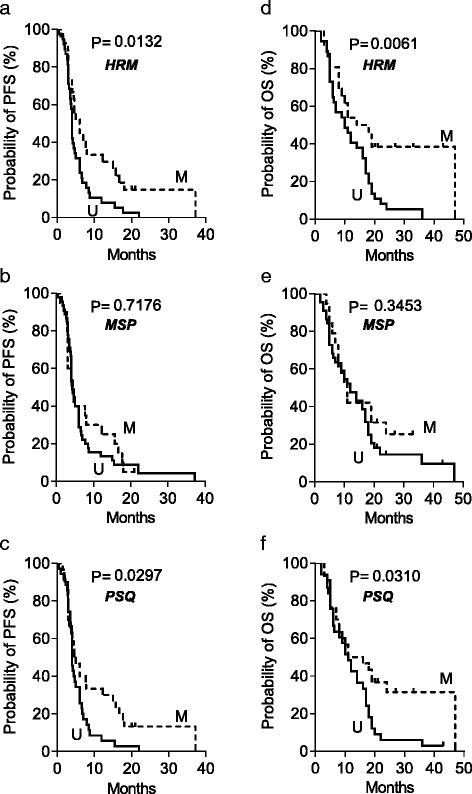
Kaplan–Meier estimates of PFS and OS according to MGMT promoter methylation status determined by HRM, MSP, and PSQ. Kaplan–Meier estimates for PFS and OS of 65 high-grade glioma patients. PFS of patients with unmethylated and methylated MGMT status, determined by HRM (**a**), MSP (**b**), and PSQ (**c**). OS of patients with unmethylated and methylated MGMT status, determined by HRM (**d**), MSP (**e**), and PSQ (**f**). Significance levels were determined by the log-rank test. *U* unmethylated; *M* methylated MGMT promoter

The association between MGMT promoter methylation and clinical outcome (using a 5 % cut-off value) comparing HRM, MSP, and PSQ was analyzed in tumor material of 65 *IDH1* wt glioma patients (seven gliomas grade III and 58 grade IV). The data are shown in Additional file 1: Table S2 for all patients in the study (including *IDH1* mutated) and in Table 2 for *IDH1* wild-type tumors only. In Table 2, we also compiled the percentage of MGMT-methylated tumors upon sex, age, and tumor grade, indicating no differences to exist between these groups. Overall, the HRM values were again more similar to PSQ than to MSP values. A comparison of Kaplan–Meier estimates of PFS using the method of HRM, MSP, and PSQ is shown in Fig. 2, panels a, b, and c, respectively. For HRM and PSQ, the difference in PFS was significant, whereas MSP did not show a significant difference. Kaplan–Meier estimates of OS of patients using the methods of HRM, MSP, and PSQ are shown in Fig. 2, panels d, e, and f, respectively (an overlay of all Kaplan–Meier curves is shown in Additional file 1: Figure S3). The data for OS also revealed an enhanced predictive value when HRM or PSQ was used compared to MSP. In a bivariate analysis, the methylation status of the MGMT promoter was correlated with PFS (*r* = 0.252, *p* = 0.042) and Karnofsky score (*r* = 0.336, *p* = 0.007). In contrast, data obtained by MSP and PSQ failed to generate significance (*p* > 0.05) both for PFS and OS.

**Table 2:** Characteristics of patients and their MGMT promoter methylation status determined by HRM, MSP, and PSQ in 65 IDH1 wt malignant gliomas

Characteristics	*n*	HRM me (%)	MSP me (%)	PSQ me (%)
All patients	65	41.5	30.8	46.2
Woman	22	40.9	31.8	40.9
Man	43	41.9	30.2	48.8
Age ≥70	27	44.4	25.9	44.4
Age <70	38	39.5	34.2	47.4
Grade III	7	42.9	42.9	42.9
Grade IV	58	41.4	29.3	46.6

**Fig. 3:**
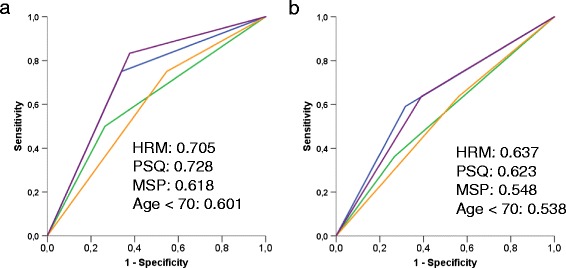
ROC curves for MGMT promoter methylation. Receiver operator characteristics (ROC) curve calculated on the basis of MGMT promoter methylation determined by MSP (*orange line*), HRM (*blue line*), PSQ (*violet line*), and age <70 (*green line*). ROC curves were calculated for (**a**) PFS ≥12 months and (**b**) OS ≥18 months. The area under the curve (AUC) corresponds to the prediction of survival, with a value of 1 indicating perfect discrimination, and a value of 0.5 no better than chance discrimination

The methods used for detection of MGMT promoter methylation were further compared by ROC analysis. ROC curves were generated to depict the sensitivity and specificity of MGMT promoter methylation status determined by HRM, MSP, PSQ as well as age <70 to predict PFS ≥12 months and OS ≥18 months. The AUC was clearly larger for HRM than for MSP both for PFS (Fig. 3a) and OS (Fig. 3b), supporting the notion that dichotomization of patients by HRM leads to less false positive and false negative results compared to MSP in predicting survival. The AUC for HRM and PSQ is nearly the same, indicating both methods provide the same discrimination accuracy.

Furthermore, univariate and multivariate Cox regression analyses were performed with the factors HRM, MSP, PSQ, sex, age <70 and grade, in order to determine independent factors for PFS and OS. In this model, HRM was found to be the only significant independent prognostic factor for OS (HR 0.473, 95 % CI 0.231–0.969, *p* = 0.041) (Table 3). Overall, the study shows that for both PFS and OS, HRM was clearly superior to MSP in discriminating between responders and non-responders and equally effective than PSQ (data are summarized in Table 4).

**Table 3:** Associations between MGMT promoter methylation status, demographic features, and grade of 65 IDH1 wt glioma patients and PFS and OS, assessed by univariate (log-rank test) and multivariate (Cox-regression) analyses

	PFS		OS	
	Univariate (*p*)	Multivariate (HR, 95% CI, *p*)	Univariate (*p*)	Multivariate (HR, 95% CI, *p*)
MSP me	0.718	1.456, 0.730–2.903, 0.285	0.345	1.270, 0.606–2.661, 0.527
PSQ me	0.030	0.729, 0.358–1.482, 0.382	0.031	0.762, 0.376–1.544, 0.450
HRM me	0.013	0.539, 0.278–1.045, 0.067	0.006	0.473, 0.231–0.969, 0.041
Sex = woman	0.742	0.958, 0.545–1.684, 0.881	0.578	0.918, 0.494–1.705, 0.786
Age <70	0.106	0.677, 0.389–1.178, 0.168	0.114	0.590, 0.331–1.052, 0.074

**Table 4:** MGMT promoter methylation status and progress and survival of patients. Promoter methylation was determined by HRM, MSP, and PSQ

Promoter status	HRM	MSP	PSQ
Methylated MGMT promoter			
Progression-free survival			
Median duration (months)	6.0 (3.64–8.36)	4.4 (3.09–5.72)	4.6 (2.88–6.32)
Rate at 6 months (%)	51.9	40.0	46.7
Overall survival			
Median duration (months)	14.0 (6.50–21.50)	11.0 (8.19–13.81)	11.0 (0.27–21.74)
Rate at 18 months (%)	48.1	40.0	46.7
Unmethylated MGMT promoter			
Progression-free survival			
Median duration (months)	4.0 (3.80–4.20)	4.2 (3.67–4.73)	4.0 (3.67–4.33)
Rate at 6 months (%)	31.6	40.0	34.3
Overall survival			
Median duration (months)	10.0 (5.03–14.97)	12.0 (7.36–16.64)	11.0 (7.62–14.38)
Rate at 18 months (%)	23.7	31.1	22.9

Numbers in parentheses are 95 % confidence intervals

## Discussion

The therapy of high-grade gliomas is based on drugs that alkylate the DNA in the O^6^-position of guanine such as temozolomide and the nitrosoureas lomustine, nimustine, and carmustine. For these drugs, MGMT is a key node in the repair of the principal toxic lesion O^6^-alkylguanine [3], determining the level of drug resistance and being a decisive factor in identifying responders and non-responders [10, 11]. The determination of MGMT activity requires native tissue and immunohistochemistry suffers from technical limitations and inter-observer differences [32]. Therefore, the method of choice for determination of the MGMT status is analysis of the MGMT promoter methylation. Since pyrosequencing is cost-intensive, MGMT promoter methylation is usually determined by MSP in the clinical routine. The human MGMT promoter is complex, harboring more than 90 CpG sites that are subject to cytosine methylation [28, 33]. For MSP, only a few of these sites in the MGMT promoter are being used. Although the methylation of the CpG sites appear to be highly variable in tumors, methylation of these target sites corresponds well with the therapeutic response, indicating that some CpG sites have a strong impact on epigenetic silencing of MGMT [33]. Of note, >50 CpG sites in the promoter region of MGMT silenced tumors were found homogeneously methylated [34]. The region commonly investigated by MSP was reported to show a concordance of about 85 % with the MGMT mRNA expression [34]. Although, the region encompassing the most often used MSP primers shows a strong concordance with MGMT silencing compared to other areas in the promoter [28], data obtained in different laboratories on this subject are quite heterogeneous [32–36]. Also for MGMT activity, a correlation was found between MGMT promoter methylation determined by MSP, but also exceptions do exist [37] indicating the importance of other methylation sites (and/or other regulatory mechanisms) in determining the MGMT expression status. It is obvious that a method covering a larger area in the MGMT promoter than encompassed by the routinely applied MSP is desirable. Another problem associated with MSP is the quality of the amplification product, which may arise due to inefficient PCR [37, 38]. Further limitations in MGMT status determination are the heterogeneity of the tumor and contamination of the tumor sample with normal cells. This pertains, however, to any PCR-based method.

HRM is an alternative method for the discrimination between 5-methylcytosine containing and non-containing DNA sequences, based on the difference in the melting curves between methylated and unmethylated templates. Compared to MSP, the HRM method relies on methylation standards that are analyzed with unknown samples, making the method investigator independent. Furthermore, HRM is a closed-tube technique that is less expensive, faster, and less laborious then methods based on DNA sequencing, including PSQ. The results obtained are quantitative. As HRM represents a real-time PCR-based method, quality control is ensured by the amplification and melting plot. The application of HRM for MGMT promoter methylation assessment has previously been proposed [21, 39]; however, a systematic comparison using a defined cut-off threshold was not undertaken and DNA standards were not verified by other methods.

To elucidate whether HRM is a feasible and reasonable alternative to MSP in determining the MGMT promoter methylation status and predicting the high-grade glioma therapy response, we compared HRM and MSP systematically. In this study, we included also PSQ, which is regarded as the “gold standard” for methylation analysis [40]. First, we showed that HRM correlates with the MGMT activity in glioblastoma cell lines. Then, we demonstrated that 51.8, 37.3, and 54.2 % of high-grade gliomas in our collection (including IDH1-mutated tumors) were promoter methylated as determined by HRM, MSP, and PSQ, respectively, indicating HRM and PSQ provided comparable results. Finally, we compared the patient’s response with the tumor methylation status, using Kaplan–Meier estimates. The data revealed a significant difference in PFS and OS between the methylated and unmethylated MGMT promoter when HRM and PSQ was used, while for MSP no significant difference was found (Fig. 2). This indicates that HRM is superior to MSP and equal to PSQ in predicting PFS and OS of high-grade glioma patients. Additional statistical evaluation like a Cox regression model showed that HRM was the only significant independent prognostic factor for OS (HR 0.473, 95 % CI 0.231–0.969, *p* = 0.041), and ROC analysis revealed that HRM and PSQ led to less false positive and false negative grouping compared to MSP in predicting survival. Overall, for both PFS and OS, HRM was clearly superior to MSP in discriminating between responders and non-responders and equally effective to PSQ.

## Conclusions

This is, to our best knowledge, the first study that compares in a well-defined tumor collection HRM, MSP, and PSQ, defining a distinct HRM promoter methylation cut-off level relevant for prediction of tumor progression and patient survival. Since the MGMT promoter methylation status analyzed by HRM is most precise in determining the patient’s outcome, we recommend HRM as a feasible and reliable method for routine diagnostics of high-grade glioma patients.

## Additional file

Additional file 1: Figure S1. (A) Normalized melt curves in duplicates showing the melt behavior of methylation standards (red=0 %, pink=25 %, blue=50 %, green=75 %, orange=100 %) and an unknown sample (black). (B) Regression model used for MGMT promoter methylation quantification. Area under the curve (AUC) from the normalized melt curves are used and regressed to the known methylation level of the standards. The linear regression model was chosen for quantification (R² > 0.98). (C) Pyrogram of the MGMT promoter of a patient tumor sample with a mean methylation of 31.4 %. (D) Typical pyrogram obtained from a grade III patient tumor sample indicating a heterozygous G-to-A point mutation of the IDH1 gene resulting in a mutation at codon 132 (R132H). (E) Relationship between MGMT protein activity and promoter methylation (r3) in 14 GBM cell lines. **Figure S2.** (A) Pyrogram of the whole HRM (R4) assay region from a patient sample. (B) Dot-plot of the methylation values obtained in a subsample by HRM and pyrosequencing showing a high correlation of this two methods. The dotted line indicates the 95 % CI. **Figure S3.** Overlay of Kaplan-Meier estimates of PFS and OS according to MGMT promoter methylation status. Kaplan-Meier estimates for (A) PFS and (B) OS of 65 high-grade glioma patients determined by HRM (red lines), MSP (blue) and PSQ (green). The solid and the dashed lines indicate the group as being categorized unmethylated (UM) or methylated (ME), respectively. **Table S1.** Primers used for HRM and pyrosequencing. **Table S2.** *MGMT* promoter methylation status determined by HRM, MSP, and PSQ in dependence of the IDH1 status. **Table S3.** ROC curves were plotted for 15 methylation cut-off scores (1-15 %) for predicting PFS ≥ 12 months and OS ≥ 18 months. (PPTX 312 kb)

## Abbreviations

AUC: area under the curve
CCNU: chlorethyl-cyclohexyl-nitroso-urea
DMEM: Dulbecco’s modified Eagle medium
DNA: deoxyribonucleic acid
GBM: glioblastoma multiforme
HRM: high resolution melt
IDH1: isocitrate dehydrogenase 1
MGMT: O^6^-alkylguanine DNA alkyltransferase
mRNA: messenger ribonucleic acid
MSP: methylation-specific PCR
OS: overall survival
PCR: polymerase chain reaction
PFS: progression-free survival
PSQ: pyrosequencing
RFU: relative fluorescence units
ROC: receiver operating characteristic
SAM: S-Adenosylmethionin
TMZ: temozolomide
