# Supplementary material for: MGMT promoter methylation determined by HRM in comparison to MSP and pyrosequencing for predicting high-grade glioma response
Source: Clin Epigenetics. 2016 May 5;8:49. doi: 10.1186/s13148-016-0204-7 (PMC4858829; doi:10.1186/s13148-016-0204-7)
Supplement: Additional file 1: Figure S1. — (A) Normalized melt curves in duplicates showing the melt behavior of methylation standards (red=0 %, pink=25 %, blue=50 %, green=75 %, orange=100 %) and an unknown sample (black). (B) Regression model used for MGMT promoter methylation quantification. Area under the curve (AUC) from the normalized melt curves are used and regressed to the known methylation level of the standards. The linear regression model was chosen for quantification (R² > 0.98). (C) Pyrogram of the MGMT promoter of a patient tumor sample with a mean methylation of 31.4 %. (D) Typical pyrogram obtained from a grade III patient tumor sample indicating a heterozygous G-to-A point mutation of the IDH1 gene resulting in a mutation at codon 132 (R132H). (E) Relationship between MGMT protein activity and promoter methylation (r3) in 14 GBM cell lines. Figure S2. (A) Pyrogram of the whole HRM (R4) assay region from a patient sample. (B) Dot-plot of the methylation values obtained in a subsample by HRM and pyrosequencing showing a high correlation of this two methods. The dotted line indicates the 95 % CI. Figure S3. Overlay of Kaplan-Meier estimates of PFS and OS according to MGMT promoter methylation status. Kaplan-Meier estimates for (A) PFS and (B) OS of 65 high-grade glioma patients determined by HRM (red lines), MSP (blue) and PSQ (green). The solid and the dashed lines indicate the group as being categorized unmethylated (UM) or methylated (ME), respectively. Table S1. Primers used for HRM and pyrosequencing. Table S2. MGMT promoter methylation status determined by HRM, MSP, and PSQ in dependence of the IDH1 status. Table S3. ROC curves were plotted for 15 methylation cut-off scores (1-15 %) for predicting PFS ≥ 12 months and OS ≥ 18 months. (PPTX 312 kb) [file 13148_2016_204_MOESM1_ESM.pptx]

## Slide 1
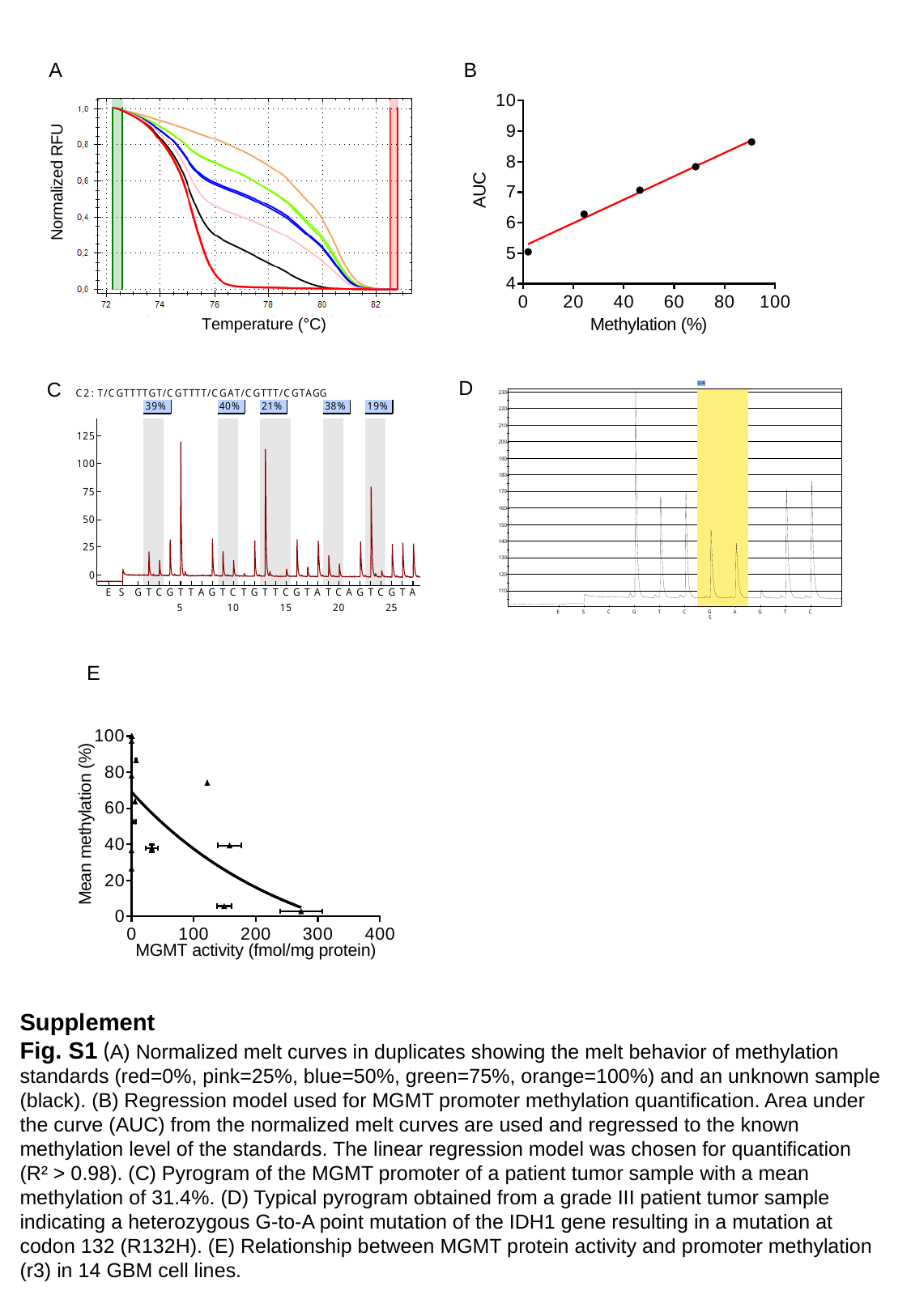

A
B
D
C
E
Supplement
Fig. S1 (A) Normalized melt curves in duplicates showing the melt behavior of methylation standards (red=0%, pink=25%, blue=50%, green=75%, orange=100%) and an unknown sample (black). (B) Regression model used for MGMT promoter methylation quantification. Area under the curve (AUC) from the normalized melt curves are used and regressed to the known methylation level of the standards. The linear regression model was chosen for quantification (R² > 0.98). (C) Pyrogram of the MGMT promoter of a patient tumor sample with a mean methylation of 31.4%. (D) Typical pyrogram obtained from a grade III patient tumor sample indicating a heterozygous G-to-A point mutation of the IDH1 gene resulting in a mutation at codon 132 (R132H). (E) Relationship between MGMT protein activity and promoter methylation (r3) in 14 GBM cell lines.

## Slide 2
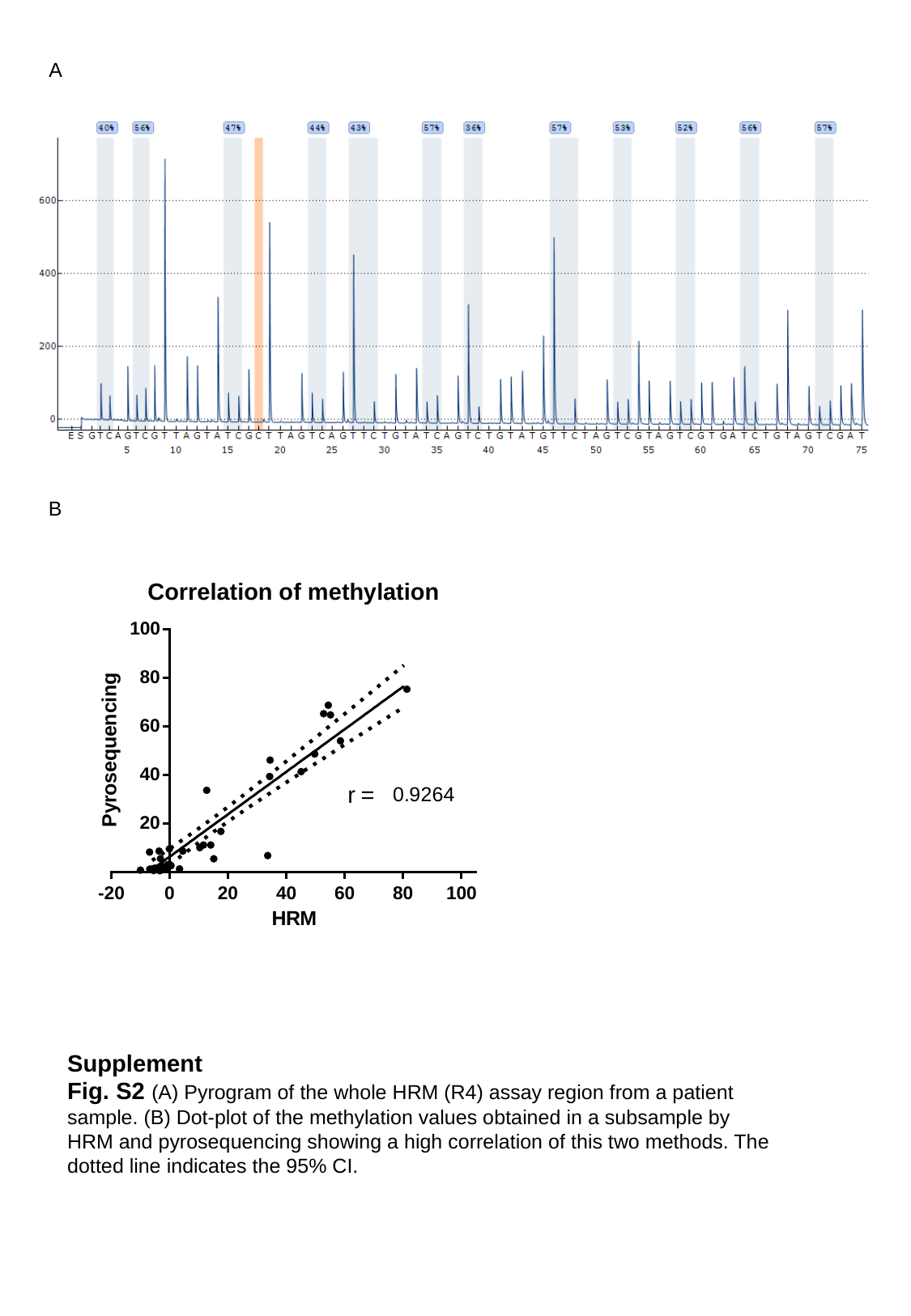

A
B
Supplement
Fig. S2 (A) Pyrogram of the whole HRM (R4) assay region from a patient sample. (B) Dot-plot of the methylation values obtained in a subsample by HRM and pyrosequencing showing a high correlation of this two methods. The dotted line indicates the 95% CI.

## Slide 3
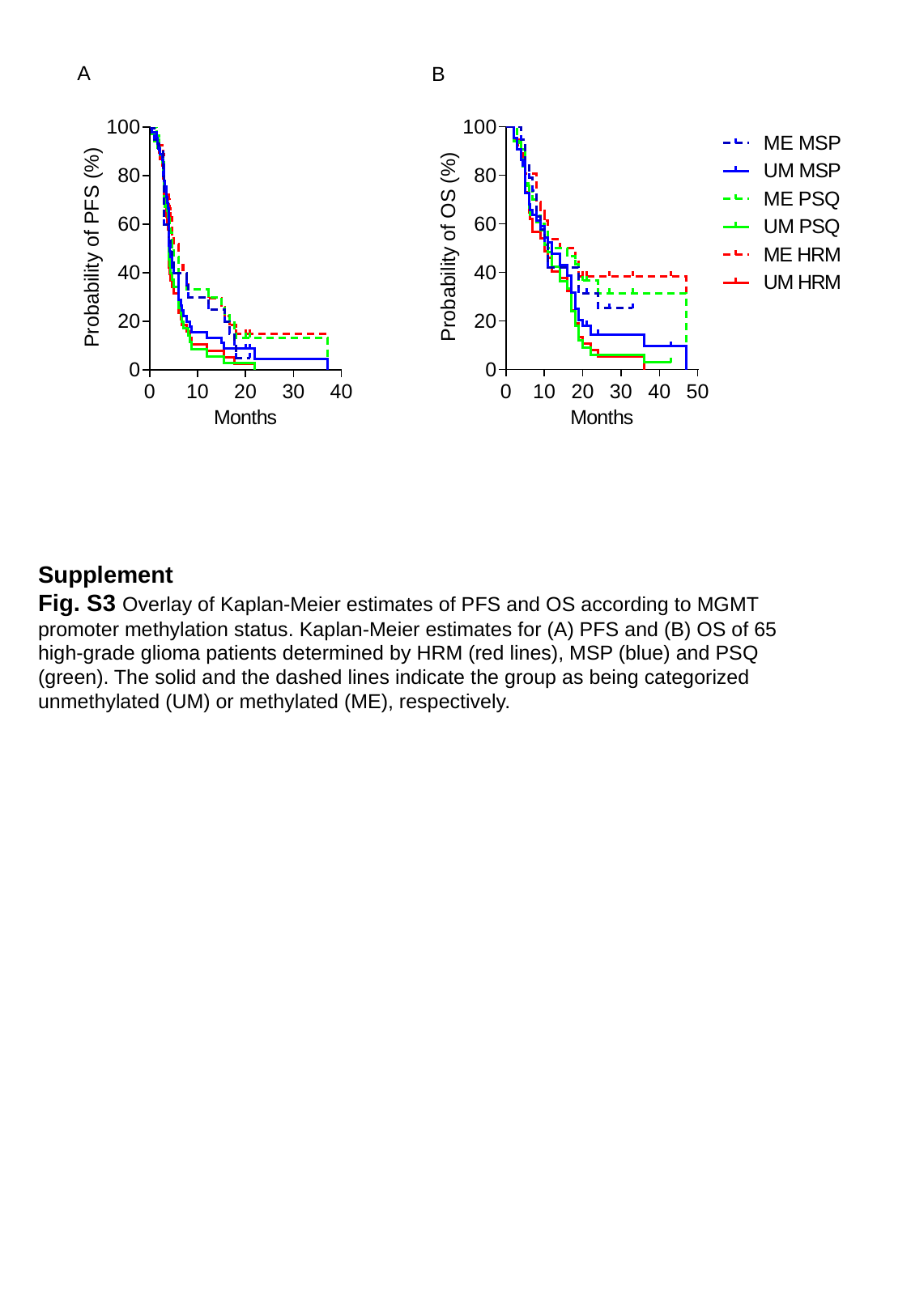

A
B
Supplement
Fig. S3 Overlay of Kaplan-Meier estimates of PFS and OS according to MGMT promoter methylation status. Kaplan-Meier estimates for (A) PFS and (B) OS of 65 high-grade glioma patients determined by HRM (red lines), MSP (blue) and PSQ (green). The solid and the dashed lines indicate the group as being categorized unmethylated (UM) or methylated (ME), respectively.

## Slide 4
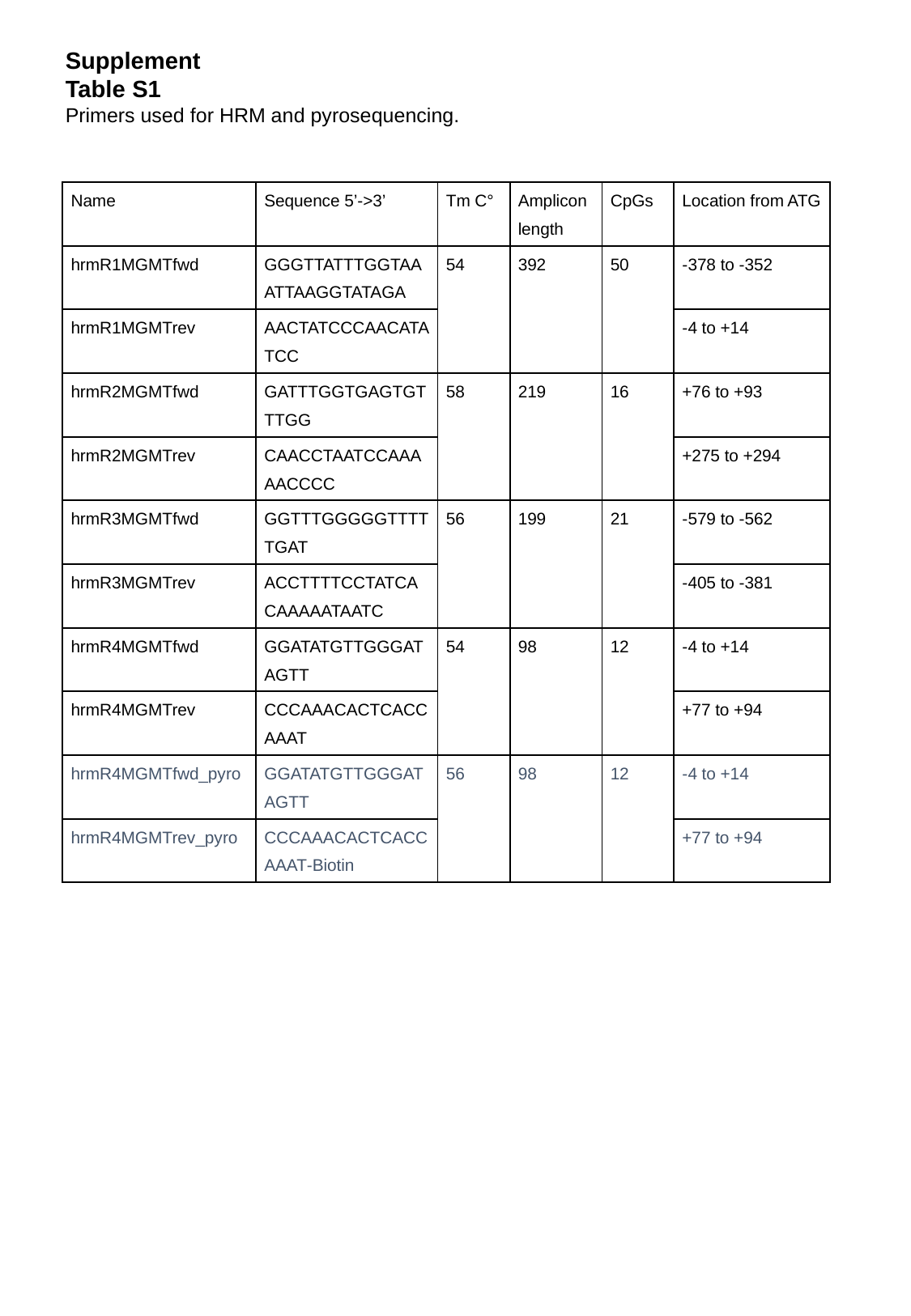

Supplement
Table S1
Primers used for HRM and pyrosequencing.
| Name | Sequence 5’->3’ | Tm C° | Amplicon length | CpGs | Location from ATG |
| --- | --- | --- | --- | --- | --- |
| hrmR1MGMTfwd | GGGTTATTTGGTAAATTAAGGTATAGA | 54 | 392 | 50 | -378 to -352 |
| hrmR1MGMTrev | AACTATCCCAACATATCC | | | | -4 to +14 |
| hrmR2MGMTfwd | GATTTGGTGAGTGTTTGG | 58 | 219 | 16 | +76 to +93 |
| hrmR2MGMTrev | CAACCTAATCCAAAAACCCC | | | | +275 to +294 |
| hrmR3MGMTfwd | GGTTTGGGGGTTTTTGAT | 56 | 199 | 21 | -579 to -562 |
| hrmR3MGMTrev | ACCTTTTCCTATCACAAAAATAATC | | | | -405 to -381 |
| hrmR4MGMTfwd | GGATATGTTGGGATAGTT | 54 | 98 | 12 | -4 to +14 |
| hrmR4MGMTrev | CCCAAACACTCACCAAAT | | | | +77 to +94 |
| hrmR4MGMTfwd\_pyro | GGATATGTTGGGATAGTT | 56 | 98 | 12 | -4 to +14 |
| hrmR4MGMTrev\_pyro | CCCAAACACTCACCAAAT-Biotin | | | | +77 to +94 |

## Slide 5
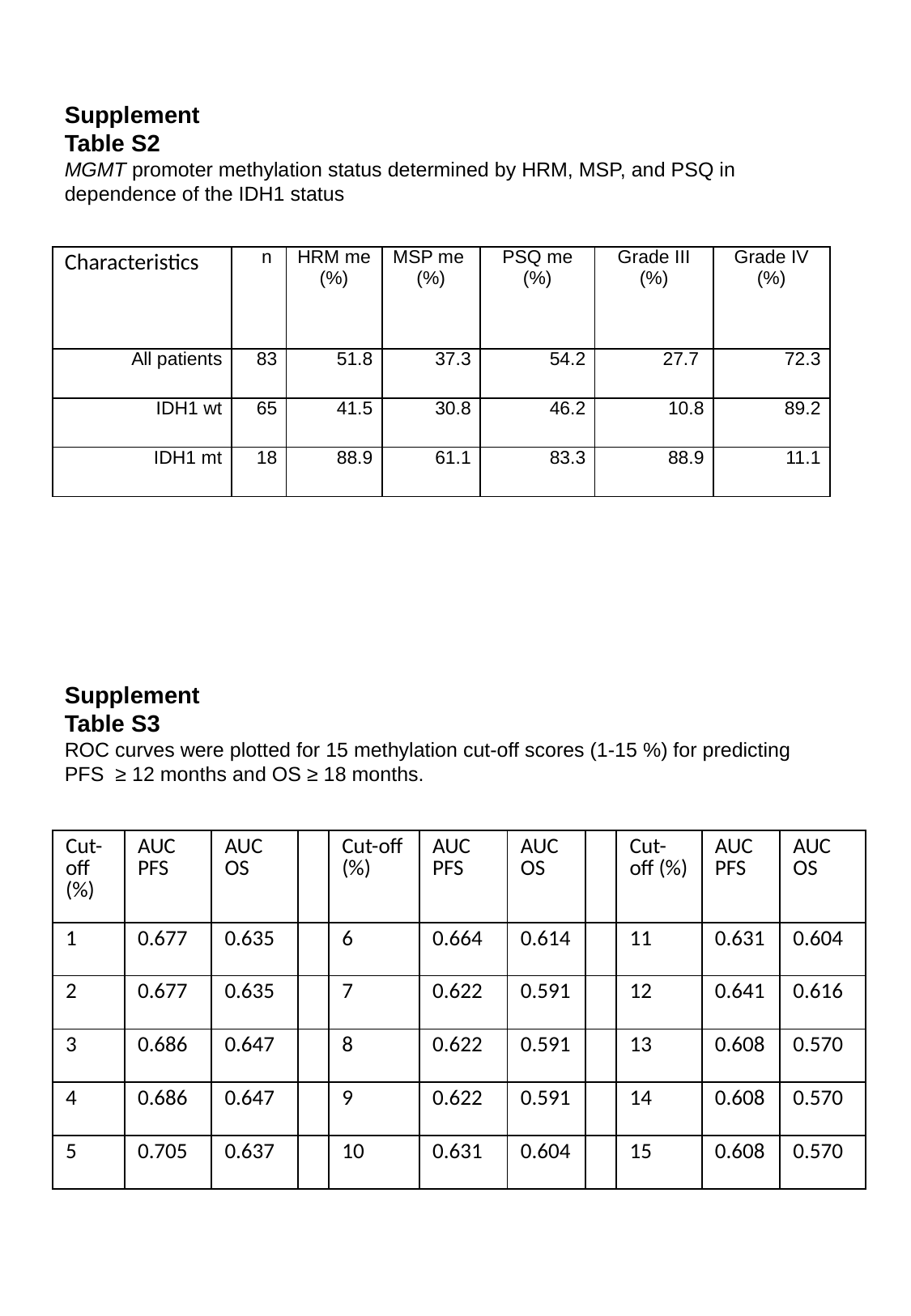

Supplement
Table S2
MGMT promoter methylation status determined by HRM, MSP, and PSQ in dependence of the IDH1 status
| Characteristics | n | HRM me (%) | MSP me (%) | PSQ me (%) | Grade III (%) | Grade IV (%) |
| --- | --- | --- | --- | --- | --- | --- |
| All patients | 83 | 51.8 | 37.3 | 54.2 | 27.7 | 72.3 |
| IDH1 wt | 65 | 41.5 | 30.8 | 46.2 | 10.8 | 89.2 |
| IDH1 mt | 18 | 88.9 | 61.1 | 83.3 | 88.9 | 11.1 |
Supplement
Table S3
ROC curves were plotted for 15 methylation cut-off scores (1-15 %) for predicting PFS ≥ 12 months and OS ≥ 18 months.
| Cut-off (%) | AUC PFS | AUC OS | | Cut-off (%) | AUC PFS | AUC OS | | Cut-off (%) | AUC PFS | AUC OS |
| --- | --- | --- | --- | --- | --- | --- | --- | --- | --- | --- |
| 1 | 0.677 | 0.635 | | 6 | 0.664 | 0.614 | | 11 | 0.631 | 0.604 |
| 2 | 0.677 | 0.635 | | 7 | 0.622 | 0.591 | | 12 | 0.641 | 0.616 |
| 3 | 0.686 | 0.647 | | 8 | 0.622 | 0.591 | | 13 | 0.608 | 0.570 |
| 4 | 0.686 | 0.647 | | 9 | 0.622 | 0.591 | | 14 | 0.608 | 0.570 |
| 5 | 0.705 | 0.637 | | 10 | 0.631 | 0.604 | | 15 | 0.608 | 0.570 |
